# Relationship between a gum‐chewing routine and oral, physical, and cognitive functions of community‐dwelling older adults: A Kashiwa cohort study

**DOI:** 10.1111/ggi.14757

**Published:** 2023-12-06

**Authors:** Jun Kawamura, Tomoki Tanaka, Susumu Kanno, Kenji Osawa, Kazuto Okabayashi, Hirohiko Hirano, Maki Shirobe, Miyuki Nagatani, Bo‐Kyung Son, Weida Lyu, Katsuya Iijima

**Affiliations:** ^1^ Institute of Gerontology The University of Tokyo Tokyo Japan; ^2^ Central Research Laboratory LOTTE CO., LTD. Tokyo Japan; ^3^ Tokyo Metropolitan Institute for Geriatrics and Gerontology Tokyo Japan; ^4^ Institute for Future Initiatives The University of Tokyo Tokyo Japan; ^5^ Graduate School of Medicine The University of Tokyo Tokyo Japan

**Keywords:** cognitive function, community‐dwelling older adults, frailty, gum, oral frailty

## Abstract

**Aim:**

As associations between oral function and general health have been reported in community‐dwelling older adults, easily implementable preventive measures are urgently required. We focused on the health benefits of gum chewing, as no studies have been carried out on the impact of gum‐chewing routines on the health of older adults. This cross‐sectional study aimed to determine whether the gum‐chewing routine is associated with oral, physical and cognitive functions in community‐dwelling older adults.

**Methods:**

This study included 1617 community‐dwelling older participants in a health survey carried out in 2021. The gum‐chewing routine and weekly chewing time were assessed using a self‐administered questionnaire. The outcome measures, including actual measurements of oral function, physical function, cognitive function, dietary intake and lifestyle, were evaluated using self‐administered questionnaires or health surveys.

**Results:**

We analyzed 1474 (mean age 76.1 ± 5.8 years, 45% women) participants for whom all data were not missing, and 14% of them had a gum‐chewing routine for more than 30 min weekly. Oral functions were significantly higher in older adults with a gum‐chewing routine, and there were substantially fewer participants with oral frailty (adjusted odds ratio 0.581, 95% confidence interval 0.340–0.993). Additionally, cognitive and physical functions, including grip strength, were significantly higher in the gum‐chewing routine group.

**Conclusions:**

Community‐dwelling older adults with a gum‐chewing routine have higher oral, physical and cognitive functions. These findings indicate that a gum‐chewing routine might contribute to maintaining oral function and preventing frailty. **Geriatr Gerontol Int 2024; 24: 68–74**.

## Graphical abstract

We focused on the health benefits of gum chewing, as no studies have been carried out on the impact of gum‐chewing routines on the health of older adults. We analyzed 1474 (mean age 76.1 ± 5.8 years, 45% women) participants for whom all data were not missing, and 14% of them had a gum‐chewing routine for more than 30 min weekly. Oral, physical and cognitive functions were significantly higher in older adults with a gum‐chewing routine.

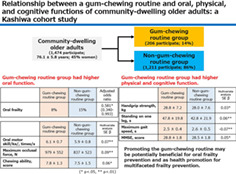

## Introduction

As the older population is increasing worldwide, extending a healthy life is becoming more crucial. In developed countries, extending healthy life expectancy is becoming highly important, and specific measures are required to prevent functional decline in old age.

Recent studies have shown a strong association between oral function and general health status.^1^ The relationship between occlusal status and medical expenses has also been shown, suggesting the impact of oral function preservation on national budgets.^2^ Consequently, momentum regarding the maintenance of oral function is growing.

Oral exercise is used to maintain and improve oral function. Various oral exercises have been developed and performed in local communities. However, making oral exercises a habit is surprisingly difficult.

Another known method for maintaining and improving oral function is gum‐chewing training. Gum‐chewing training has been shown to significantly improve occlusal force and tongue pressure in adults.^3^, ^4^, ^5^ Reportedly, gum chewing increases cerebral blood flow and enhances cognitive functions, such as attention and cognitive memory, in older adults.^6^, ^7^ An intervention study in older adults confirmed that gum‐chewing training enhanced chewing and balance abilities, including the time to lift one leg with open eyes.^8^

Based on these previous studies, we hypothesized that including gum‐chewing training as a routine might be useful in maintaining and improving oral, physical, and cognitive functions in older adults. Gum is a food that can be purchased at nearby stores and is accessible to consumers. Therefore, gum chewing can readily become a routine, and if it contributes to the health of older adults, it is straightforward for them to include it in their daily lives. However, to the best of our knowledge, no studies have investigated the impact of gum‐chewing routines on the health of older adults.

Therefore, here, we carried out a cross‐sectional analysis of the relationship between the gum‐chewing routine and oral, cognitive, and physical functions in community‐dwelling older adults in Japan to elucidate whether the gum‐chewing routine might contribute to the health of older adults.

## Methods

### Study setting and participants

In the present cross‐sectional study, we analyzed data from the Kashiwa study. Overall, 1617 participants were included in the sixth round of a prospective cohort study of older adults aged ≥65 years living in Kashiwa City. They were randomly selected from the basic resident register of Kashiwa City as follows: 442 people were selected during the first wave in 2012 and have continued to participate, and 1175 new individuals were chosen from the second wave in 2021. No significant difference was found in the basic data used for the sampling. All participants agreed to participate in the survey. The inclusion criteria were as follows: (i) community‐dwelling older adults (aged ≥65 years); and (ii) lack of long‐term care insurance certification at the random selection. The exclusion criterion was missing data for variable items.

Surveys were carried out at neighborhood and health centers in the city between September and November 2021. All inspectors received the prescribed training to calibrate their assessment methods.

The ethics committee of the University of Tokyo Life Science Research Center approved this study's protocol (#21‐192). Written informed consent was obtained from all participants. This study was carried out following the principles of the Declaration of Helsinki.

### Measures

The detailed requirements for the measurement status are summarized in the Data S1 Methods section.

### Gum‐chewing routine

Here, the gum‐chewing routine was assessed using an original self‐administered questionnaire. Participants responded to the question, “Do you have a routine of chewing gum?” with six different frequencies; those who responded to having a gum‐chewing habit were also asked how many minutes per week they chewed gum in total; the median time of chewing was determined for those who responded to having the gum‐chewing habit, and those who chewed more than that value were considered in the gum‐chewing routine group.

### Basic attributes

We assessed basic attributes, including age, sex, body mass index (BMI), living arrangements, education duration, number of medications, physical activity, social network scores, health literacy and frequency of visits to the dentist. The data were obtained from medical interviews, actual measurements and self‐administered questionnaires.

The InBody430 (InBody Japan, Tokyo, Japan) was used to measure the participants' BMI; however, those who could not use the InBody were asked to self‐report their weight, and the BMI was calculated. Physical activity was assessed using the Global Physical Activity Questionnaire, based on leisure‐ and work‐time activity involving moderate‐to‐vigorous intensity and travel‐related activity.^9^ Social network scores were evaluated using the Lubben Social Network Scale‐6 (LSNS‐6), which assesses the number of family members/friends with whom one has social contact and receives social support; it is a widely used validated social isolation screening tool among community‐dwelling older adults.^10^ The scale ranges from 0 to 30, with a higher score indicating a stronger social support network. Health literacy was evaluated using the Communicative and Critical Health Literacy scale.^11^ The frequency of visits to the dentist was converted into an ordinal scale using a self‐administered questionnaire, where respondents were asked to choose “1. At least once every 3 months,” “2. At least once every 6 months,” “3. At least once a year,” “4. When there are symptoms, such as toothache,” or “5. Rarely”.^12^

### Oral function

The oral function was assessed based on the dental status, oral function and subjective ratings. Dentists and dental hygienists with experience in clinical practice under the supervision of a dentist evaluated all measurements. The dentists and the dental hygienists were pre‐trained to ensure the uniformity of the judgment criteria.

Dental status: this included the number of remaining teeth, the number of functional teeth and the tongue coating index.

Oral function: maximum occlusal force, chewing ability, articulatory oral motor skill (“pa,” “ta” and “ka”), tongue pressure and oral moistness.

Subjective assessments: difficulties in eating hard foods, difficulties in swallowing tea or soup and experience of having a dry mouth were evaluated using the questionnaire.

Additionally, oral frailty was defined as three or more of the following six items: (i) the number of natural teeth, (ii) chewing ability, (iii) articulatory oral motor skill for “ta,” (iv) tongue pressure, (v) subjective difficulty in eating tough foods, and (vi) subjective difficulty in swallowing.^1^

### Frailty

Frailty was evaluated using the Kihon Checklist (KCL).^13^ Frailty was defined as eight or more items, and the number of items on the KCL was also assessed.

### Physical function

Physical functions measured included grip strength (grip dynamometer), one‐leg stand with eyes open for 60 s, Timed “Up and Go” test, normal gait speed and maximum gait speed.

### Cognitive function

Cognitive function was assessed using the Mini‐Mental State Examination.^14^

### Nutritional status

Food intake was assessed using a brief self‐administered diet history questionnaire.^15^ The density method was used for correction. Here, we specifically evaluated the nutrients that had been studied regarding chewing ability in previous studies.^16^, ^17^

### Statistical analysis

All statistical analyses were carried out using IBM Statistical Package for Social Sciences for Windows, version 28.0 (IBM Corporation, Armonk, NY, USA), and the statistical significance was set at *P* < 0.05.

We compared the characteristics between the gum‐chewing routine and the non‐gum‐chewing routine groups. Continuous and categorical variables were evaluated using the Mann–Whitney *U*‐test and χ^2^‐test, respectively.

As we also hypothesized that the gum‐chewing routine was associated with health in older adults, a binomial logistic regression model was used to calculate odds ratios and 95% confidence intervals for the outcomes indicated by the categorical variables. For outcomes presented as continuous variables, single and multiple regression analyses were used to calculate partial regression coefficients, adjusted for degrees of freedom.

For the relationship between gum‐chewing routine and oral function, we adjusted for the following covariates: age, sex, BMI, living arrangements, education duration, number of medications, health literacy, LSNS‐6 scores and frequency of visits to the dental clinic as confounding factors. In the relationship between gum‐chewing routine and physical function, cognitive function, and nutritional intake status, covariates, such as age, sex, BMI, living arrangements, education duration, number of medications, health literacy and LSNS‐6 scores, were adjusted for as confounding factors.

## Results

Of the 1617 participants, 143 were excluded owing to missing data, and a cross‐sectional analysis was carried out with the remaining 1474 (mean age 76.1 ± 5.8 years, 45% women). A flow diagram of the survey is shown in Figure S1. Of the respondents, 350 (23.7%) indicated that they had a gum‐chewing habit. Those with gum‐chewing habits were asked about their weekly gum‐chewing time, with the shortest and longest being 1 min/week and 3360 min/week, respectively, with a median of 30 min. Therefore, 206 (14.0%) participants with a gum‐chewing time of 30 min/week or longer were categorized into the gum‐chewing routine group. A comparison of the characteristics of the gum‐chewing routine and non‐gum‐chewing routine groups is presented in Table 1.

**Table 1:** Comparison of characteristics of the gum‐chewing routine group and non‐gum‐chewing routine group

Factors		Overall	Gum‐chewing routine group	Non‐gum‐chewing routine group	*P* ^†^
No. participants		1474	206	1268	
Basic attributes	Age (years)	76.1 ± 5.8	75.5 ± 5.4	76.2 ± 5.8	0.184
	Sex (women)	661 (44.8%)	95 (46.1%)	566 (44.6%)	0.692
	Body mass index (kg/m^2^)	23.5 ± 2.7/22.2 ± 3.1	23.7 ± 2.6/22.1 ± 3.1	23.4 ± 2.8/22.2 ± 3.1	0.984
	Living alone	209 (14.2%)	29 (14.1%)	180 (14.2%)	0.964
	Education (years)	13.6 ± 2.9	13.6 ± 2.8	13.6 ± 3.1	0.571
	No. medications (*n*)	2.9 ± 2.7	2.8 ± 2.5	2.9 ± 2.7	0.925
	GPAQ (Week Mets)	3930 ± 4560/3520 ± 4640	4380 ± 4680/3290 ± 3600	3860 ± 4540/3560 ± 4790	0.419
	LSNS‐6 score	14.1 ± 5.6	14.3 ± 5.7	14.1 ± 5.5	0.689
	CCHL score	19.9 ± 3.3	20.0 ± 3.3	19.9 ± 3.3	0.713
	Frequency of visits to the dentist	2.3 ± 1.3	2.2 ± 1.2	2.3 ± 1.4	0.612
Chronic diseases	Diabetes mellitus	184 (12.5%)	19 (9.2%)	165 (13.0%)	0.127
	Hypertension	672 (45.6%)	90 (43.7%)	582 (45.9%)	0.555
	Osteoporosis	180 (12.2%)	26 (12.6%)	154 (12.2%)	0.853
	Stroke	76 (5.2%)	11 (5.3%)	65 (5.1%)	0.904
	Dyslipidemia	591 (40.1%)	85 (41.3%)	506 (39.9%)	0.712
	Malignant neoplasm	294 (19.9%)	42 (20.4%)	252 (19.9%)	0.864
	Heart diseases	240 (16.3%)	43 (20.9%)	197 (15.5%)	0.054

*Note*: Data are shown as the mean (±standard deviations) for quantitative measures, and as the number of participants (percentages) for all qualitative measures.

CCHL, Communicative and Critical Health Literacy scale; GPAQ, Global Physical Activity Questionnaire; LSNS‐6, Lubben Social Network Scale‐6.

^†^ Differences in variables among those with and without gum‐chewing routine were analyzed using the χ^2^‐test for categorical variables and Mann–Whitney *U*‐test for continuous variables.

No significant differences were observed between the gum‐chewing and non‐gum‐chewing groups regarding health literacy scores and the number of medications taken.

Based on the hypothesis, a binomial logistic regression analysis was carried out to determine whether the gum‐chewing routine affected the health status of the participants (Table 2).

**Table 2:** Oral and frailty status (categorical value) between the gum‐chewing routine group and the non‐gum‐chewing routine group

Factors (dependent variables)	Overall (*n* = 1474)	Gum‐chewing routine group (*n* = 206)	Non‐gum‐chewing routine group (*n* = 1268)	Non‐Gum‐chewing routine group	Gum‐chewing routine group	
				OR	Crude OR (95% CI)^†^	aOR (95% CI)^‡^
Oral status						
Difficulties in eating hard foods (yes)	265 (18.0%)	23 (11.2%)	242 (19.1%)	1.00 (reference)	0.53 (0.34–0.84)**	0.55 (0.34–0.87)*
Difficulties in swallowing tea or soup (yes)	312 (21.2%)	48 (23.3%)	264 (20.8%)	1.00 (reference)	1.16 (0.81–1.64)	1.18 (0.83–1.68)
Experience having a dry mouth (yes)	371 (25.2%)	57 (27.7%)	314 (24.8%)	1.00 (reference)	1.16 (0.84–1.62)	1.23 (0.88–1.73)
Frailty						
Oral frailty	205 (13.9%)	17 (8.3%)	188 (14.8%)	1.00 (reference)	0.52 (0.31–0.87)*	0.58 (0.34–0.99)*
Frailty using by KCL	336 (22.8%)	36 (17.5%)	300 (23.7%)	1.00 (reference)	0.68 (0.47–1.00)*	0.60 (0.47–1.06)

*Note*: Data are shown as the number of participants (percentages).

Abbreviations: aOR, adjusted odds ratio; CI, confidence interval; KCL, Kihon Checklist; OR, odds ratio.

^†^ Results of binomial logistic regression analysis when with/without gum‐chewing routine was used as a covariate, and each factor was used as a dependent variable (*P < 0.05, **P< 0.01).

^‡^ Adjusted model included as covariates: age, sex, body mass index, living arrangements, education duration, number of medications, Lubben Social Network Scale‐6 scores, and Communicative and Critical Health Literacy scale scores. For only analysis of oral status and oral frailty, and the frequency of visits to the dentist was also used as a covariate. (*P < 0.05, **P < 0.01).

After considering various factors, including age and sex, the corresponding percentages for whether they had less difficulty eating hard foods than they had 6 months earlier were significantly lower in the gum‐chewing routine group. Additionally, the prevalence of oral frailty was considerably lower. Notably, the number of participants in the gum‐chewing routine group was significantly lower regarding the number of remaining teeth, tongue pressure and subjective masticatory ability (Table S1).

As shown in Table 2, it was evident that there were likely to be differences in some statuses between the gum‐chewing and non‐gum‐chewing routine groups; therefore, we also analyzed each of the measured statuses.

Table 3 shows the results of the single and multiple regression analyses for the gum‐chewing routine and each oral and frail status. The results showed that the gum‐chewing routine group had a higher number of remaining teeth. Furthermore, despite no difference in the number of functional teeth, maximum occlusal force, chewing ability and oral motor skill “ka” were significantly higher. The number of applicable oral frailty symptoms and hits on the KCL, which evaluated overall oral function and physical frailty, respectively, were also significantly lower.

**Table 3:** Oral and frailty status (continuous value) between the gum‐chewing routine group and the non‐gum‐chewing routine group

Factors (objective variables)	Overall (*n* = 1474)	Gum‐chewing routine group (*n* = 206)	Non‐gum‐chewing routine group (*n* = 1268)	Gum‐chewing routine (explanatory variable)			
				Univariate analysis^†^		Multivariate analysis^‡^	
				SE *β*	*P*	SE *β*	*P*
Oral status							
No. remaining teeth (*n*)	22.5 ± 7.3	25.1 ± 4.5	22.1 ± 7.6	0.14	< 0.001**	0.13	< 0.001**
No. functional teeth (*n*)	27.1 ± 2.8	27.2 ± 2.8	27.1 ± 2.8	0.02	0.544	0.01	0.655
TCI (%)	34.0 ± 23.0	35.3 ± 23.6	33.7 ± 22.9	0.02	0.375	0.03	0.286
Maximum occlusal force (N)	944 ± 568/750 ± 455	1100 ± 581/833 ± 479	918 ± 562/736 ± 450	0.09	< 0.001**	0.09	< 0.001**
Chewing ability, score	7.6 ± 1.4/7.5 ± 1.5	8.0 ± 1.3/7.6 ± 1.4	7.6 ± 1.5/7.5 ± 1.5	0.07	0.006**	0.06	0.013*
Oral motor skill/pa/ (times/s)	6.4 ± 0.8/6.5 ± 0.7	6.5 ± 0.7/6.6 ± 0.6	6.4 ± 0.8/6.5 ± 0.7	0.05	0.083	0.04	0.167
Oral motor skill/ta/ (times/s)	6.4 ± 0.9/6.5 ± 0.7	6.5 ± 0.8/6.6 ± 0.7	6.4 ± 0.9/6.4 ± 0.7	0.05	0.049*	0.04	0.114
Oral motor skill/ka/ (times/s)	5.9 ± 0.9/6.1 ± 0.7	6.0 ± 0.7/6.3 ± 0.7	5.8 ± 0.9/6.1 ± 0.7	0.08	0.003**	0.07	0.010**
Tongue pressure (kPa)	33.2 ± 7.7/31.4 ± 7.1	34.7 ± 7.5/31.7 ± 6.4	32.9 ± 7.7/31.4 ± 7.3	0.05	0.050*	0.04	0.101
Oral wettability	27.8 ± 5.7/27.2 ± 2.6	27.8 ± 4.9/27.1 ± 3.2	27.8 ± 5.9/27.3 ± 2.5	−0.01	0.826	−0.01	0.753
Frailty							
Applicable no. oral frailty 6 items	1.2 ± 1.2	0.9 ± 1.0	1.2 ± 1.2	−0.11	< 0.001**	−0.09	< 0.001**
KCL score	5.4 ± 3.0	4.9 ± 2.7	5.5 ± 3.1	−0.06	0.024*	−0.05	0.040*

*Note*: Data are shown as means (±standard deviations). Some factors are indicated by males/females.

Abbreviations: KCL, Kihon Checklist; SE, standard partial regression coefficient; TCI, tongue coating index.

^†^ Results of univariate analysis when with/without gum‐chewing routine was used as a covariate, and each factor was used as a dependent variable (*P < 0.05, **P < 0.01).

^‡^ Adjusted model included as covariates: with/without gum‐chewing routine, age, sex, body mass index, living arrangements, education duration, number of medications, Lubben Social Network Scale‐6 scores, and Communicative and Critical Health Literacy scale scores, and frequency of visits to the dentist (*P < 0.05, **P < 0.01).

Table 4 shows the results of the single and multiple regression analyses for the gum‐chewing routine and each physical and cognitive status. The results showed that the gum‐chewing routine group had higher handgrip strength, a longer time standing on one leg, faster maximum gait speed and significantly higher Mini‐Mental State Examination scores.

**Table 4:** Physical and cognitive status between the gum‐chewing routine group and the non‐gum‐chewing routine group

Factors (objective variables)	Overall (*n* = 1474)	Gum‐chewing routine group (*n* = 206)	Non‐gum‐chewing routine group (*n* = 1268)	Gum‐chewing routine (explanatory variable)			
				Univariate analysis^†^		Multivariate analysis^‡^	
				SE *β*	*P*	SE *β*	*P*
Handgrip strength, kg	33.1 ± 6.0/22.1 ± 4.0	34.0 ± 5.2/22.8 ± 3.8	33.0 ± 6.1/21.9 ± 4.0	0.04	0.158	0.03	0.050*
Standing on one leg (s)	43.1 ± 21.9/44.1 ± 21.5	47.3 ± 20.3/48.4 ± 19.2	42.4 ± 22.0/43.4 ± 21.7	0.08	0.002**	0.06	0.009**
TUG (s)	5.8 ± 1.3/6.1 ± 1.4	5.7 ± 1.0/5.9 ± 1.2	5.9 ± 1.4/6.2 ± 1.5	−0.05	0.040*	−0.03	0.127
Normal gait speed (s)	3.9 ± 0.8/3.9 ± 0.8	3.8 ± 0.6/3.8 ± 0.6	4.0 ± 0.9/3.9 ± 0.8	−0.06	0.029*	−0.04	0.073
Maximum gait speed (s)	2.5 ± 0.5/2.7 ± 0.5	2.4 ± 0.4/2.6 ± 0.5	2.5 ± 0.5/2.7 ± 0.5	−0.08	0.002**	−0.07	0.005**
MMSE score	28.5 ± 1.8	28.8 ± 1.8	28.5 ± 1.8	0.06	0.020*	0.05	0.048*

*Note*: Data are shown as means (±standard deviations). Some factors are indicated by males/females.

Abbreviations: MMSE, Mini‐Mental State Examination; SE, standard partial regression coefficient; TUG, Timed Up and Go test.

^†^ Results of binomial logistic regression analysis when with/without gum‐chewing routine was used as a covariate, and each factor was used as a dependent variable (*P < 0.05, **P < 0.01).

^‡^ Adjusted model included as covariates: with/without gum‐chewing routine, age, sex, body mass index, living arrangements, education duration, number of medications, Lubben Social Network Scale‐6 scores, and Communicative and Critical Health Literacy scale scores. (*P < 0.05, **P < 0.01).

Table 5 presents the results of single and multiple regression analyses for gum‐chewing routine and nutritional status. The results showed that the gum‐chewing routine group had significantly higher protein, potassium, calcium, magnesium, phosphorus, iron, and vitamins A, D, K, B_12_ and C levels.

**Table 5:** Each nutrition status between the gum‐chewing routine group and the non‐gum‐chewing routine group

Factors (objective variables)	Overall (*n* = 1474)	Gum‐chewing routine group (*n* = 206)	Non‐gum‐chewing routine group (*n* = 1268)	Gum‐chewing routine (explanatory variable)	
				SE *β*	
				Univariate analysis^†^	Multivariate analysis^‡^
Energy (kcal)	2180 ± 640/1900 ± 580	2100 ± 570/1850 ± 490	2190 ± 650/1910 ± 590	−0.05	−0.04
Protein (g/1000 kcal)	43.3 ± 8.5	44.4 ± 8.9	43.1 ± 8.4	0.05 *	0.06 *
Fat (g/1000 kcal)	32.3 ± 6.3	32.4 ± 5.9	32.2 ± 6.3	0.01	0.01
Carbohydrate (g/1000 kcal)	123 ± 20	123 ± 19	123 ± 20	0.00	0.01
Sodium (mg/1000 kcal)	2600 ± 510	2650 ± 480	2590 ± 510	0.04	0.05
Potassium (mg/1000 kcal)	1640 ± 420	1700 ± 400	1630 ± 420	0.06*	0.06*
Calcium (mg/1000 kcal)	370 ± 110	391 ± 110	367 ± 110	0.08**	0.08**
Magnesium (mg/1000 kcal)	161 ± 33	167 ± 34	160 ± 33	0.07**	0.08**
Phosphorus (mg/1000 kcal)	670 ± 140	693 ± 140	666 ± 140	0.07**	0.07**
Iron (mg/1000 kcal)	5.1 ± 1.2	5.3 ± 1.3	5.1 ± 1.2	0.06*	0.06*
Vitamin A (μg/1000 kcal)	480 ± 280	518 ± 350	474 ± 270	0.05*	0.06*
Vitamin D (μg/1000 kcal)	10.6 ± 5.9	11.5 ± 6.5	10.4 ± 5.8	0.07*	0.07**
Vitamin K (μg/1000 kcal)	217 ± 97	230 ± 110	215 ± 96	0.05*	0.05*
Vitamin B_1_ (mg/1000 kcal)	0.5 ± 0.1	0.5 ± 0.1	0.5 ± 0.1	0.03	0.03
Vitamin B_2_ (mg/1000 kcal)	0.9 ± 0.2	0.9 ± 0.2	0.8 ± 0.2	0.04	0.04
Vitamin B_6_ (mg/1000 kcal)	0.8 ± 0.2	0.8 ± 0.2	0.8 ± 0.2	0.04	0.04
Vitamin B_12_ (μg/1000 kcal)	6.8 ± 3.3	7.3 ± 3.7	6.7 ± 3.2	0.06*	0.06*
Vitamin C (mg/1000 kcal)	77.0 ± 31.4	80.7 ± 31.7	76.4 ± 31.3	0.05	0.05*

*Note*: Data are shown as means (±standard deviations). Some factors are indicated by males/females.

Abbreviations: SE, standard partial regression coefficient.

^†^ Results of univariate analysis when with/without gum‐chewing routine was used as a covariate, and each factor was used as a dependent variable (*P < 0.05, **P < 0.01).

^‡^ Adjusted model included as covariates: with/without gum‐chewing routine, age, sex, body mass index, living arrangements, education duration, number of medications, Lubben Social Network Scale‐6 scores, and Communicative and Critical Health Literacy scale scores. (*P < 0.05, **P < 0.01).

## Discussion

The present study investigated the relationship between the gum‐chewing routine and oral, physical and cognitive functions, and nutritional status in community‐dwelling older adults. Our results showed that many older adults with gum‐chewing routines had good oral, physical and cognitive functions, and nutritional status.

The cross‐sectional analysis of this study showed a lower prevalence of oral frailty in the gum‐chewing routine group, even after considering age, sex, BMI, living alone or not, years of schooling, health literacy, type of oral medication, LSNS‐6 scores and frequency of visits to the dental clinic. Regarding oral function, subjective chewing ability (whether it was harder to eat hard foods than it was 6 months ago), the number of remaining teeth, chewing ability, bite strength and oral motor skill “ka” were significantly higher in the gum‐chewing routine group. Previous studies have reported that gum training interventions improved oral functions, including bite strength, tongue pressure and chewing ability.^3^, ^4^, ^5^, ^8^ Regarding its mechanism of action of gum training to improve oral function in previous studies, it is as effective as pure training, and the saliva secreted by chewing is influential. Therefore, the gum‐chewing routine might have led to oral training and maintenance or improvement in oral functions in the present study.

Additionally, the gum‐chewing routine group showed significantly better values for the number of hits on KCL, grip strength, standing on one leg and maximum gait speed. As no significant difference was found in the Global Physical Activity Questionnaire (Week Mets), in this case, we consider that the gum‐chewing routine group had no higher physical function due to their higher usual activity levels. Previous studies have shown a correlation between occlusal status and physical balance, and intervention trials with gum chewing have shown an improved balance ability.^8^, ^18^, ^19^ Therefore, it is reasonable to expect that the results of physical functions where balance is important, including standing on one leg, would be significantly higher in the gum‐chewing routine group. Furthermore, the gum‐chewing routine group showed higher values for muscular strength status, including grip strength. Because teeth clenching increases muscle power, it is possible that the increase in muscle power caused by clenching was stronger in the gum‐chewing routine group owing to their higher oral functions.^20^ Furthermore, the gum‐chewing routine group had better nutritional status, despite no difference in total calorie intake. This suggests that physical function has been maintained at a high level because of the high mastication ability and nutritionally balanced diet. It is also known that cerebral blood flow increases during gum chewing.^7^ Therefore, the gum‐chewing routine might have influenced physical and brain functions because of the increased cerebral blood flow.

Regarding cognitive function, the gum‐chewing routine group had significantly higher Mini‐Mental State Examination scores. The relationship between mastication ability and cognitive function has been clarified, and the association between overall oral and cognitive functions has also been established.^21^, ^22^ Because gum chewing has been shown to increase cerebral blood flow,^6^ it is reasonable to assume that cognitive function was higher in the gum‐chewing routine group.

Nutritional status was also significantly higher for several nutrients in the gum‐chewing routine group. Notably, the intake of protein and various minerals, which has been reported in previous studies to decrease when chewing ability declines, was higher in the gum‐chewing routine group. However, no significant difference was observed in caloric intake. This might be because the high oral function of the gum‐chewing routine group maintained food diversity by increasing the variety of foods they could consume.

Therefore, based on these results and previous studies,^3^, ^4^, ^5^, ^8^ it is suggested that the habit of chewing gum is useful in maintaining oral function in older adults. Furthermore, these results indicate that gum chewing affects oral, physical and cognitive functions. One of the reasons for stopping gum chewing in old age is the use of dentures; however, gum that does not stick to dentures has recently become available, and based on the benefits of the gum‐chewing routine suggested in the present study, it would be more beneficial to maintain oral functions by adopting gum‐chewing routine in daily life. Given that people with dentures have less periodontal tissue, including the periodontal ligament, than those with remaining teeth, differences might exist in the health benefits obtained; therefore, further research is desirable. However, as gum chewing can readily become a routine and gum is easy to obtain, it can influence the health of older adults and can become a more convenient health routine.

To the best of our knowledge, the present study is the first to examine the relationship between daily gum‐chewing routines and oral, physical, and cognitive functions in older adults. However, this study had some limitations. First, this was a cross‐sectional study; therefore, a clear causal relationship could not be established. Second, this study used chewing time in the questionnaire, which might have deviated from actual chewing time. Third, chewing time was not constant in the gum‐chewing routine group. Fourth, the participants in this study might be representative of healthy community‐dwelling older adults; however, further research is needed to determine whether they represent cognitively impaired people or those in long‐term care. Fifth, although this study adjusted for various confounding factors, there is a possibility that unassessed factors, including whether the patient was receiving dental treatment, might have influenced the results.

Community‐dwelling older adults with a gum‐chewing routine have better oral, physical and cognitive functions. and nutritional status. Promoting the gum‐chewing routine might benefit oral frailty prevention and health promotion for multifaceted frailty prevention. Therefore, prospective observational studies and research on the health effects of a gum‐chewing training intervention in older adults are desirable, using this study as a stepping stone.

## Disclosure statement

The authors declare no conflict of interest.

## Supporting information


**DATA S1.** Supporting Information.

## Data Availability

The data that support the findings of this study are available on request from the corresponding author. The data are not publicly available due to privacy or ethical restrictions.

## References

[1] Tanaka T , Takahashi K , Hirano H *et al*. Oral frailty as a risk factor for physical frailty and mortality in community‐dwelling elderly. J Gerontol A Biol Sci Med Sci 2018; 73: 1661–1667. 29161342 10.1093/gerona/glx225

[2] Ishikawa M , Yasuda T , Takase N . Relationship between Oral health conditions and medical care expenditure based on receipt information. Jpn J Dent Prac Admin 2021; 56: 32–41.

[3] Shirai M , Kawai N , Hichijo N *et al*. Effects of gum chewing exercise on maximum bite force according to facial morphology. Clin Exp Dent Res 2018; 4: 48–51. 29744215 10.1002/cre2.102 PMC5893462

[4] Masumoto N , Yamaguchi K , Fujimoto S . Daily chewing gum exercise for stabilizing the vertical occlusion. J Oral Rehabil 2009; 36: 857–863. 19845836 10.1111/j.1365-2842.2009.02010.x

[5] Takahashi M , Satoh Y . Effects of gum chewing training on oral function in normal adults: part 1 investigation of perioral muscle pressure. J Dent Sci 2019; 14: 38–46. 30988878 10.1016/j.jds.2018.11.002 PMC6445978

[6] Nagashima S , Kimoto K , Ono Y *et al*. The effect of masticatory behaviour on generalized attention in heathy volunteers. Psychogeriatrics 2020; 20: 254–261. 31881113 10.1111/psyg.12493

[7] Sasaguri K , Umeda Y , Sagisaka E , Kubo K . Chewing‐induced enhancement of cognitive memory associated with the hippocampus and the prefrontal cortex in the elderly. J Health Educ 2012; 28: 179–191.

[8] Nakazawa M , Mori H , Handa J *et al*. Simple training for maintaining and improving chewing ability. Jpn J Gerodontol 2018; 33: 63–69.

[9] Bull FC , Maslin TS , Armstrong T . Global physical activity questionnaire (Gpaq): nine country reliability and validity study. J Phys Act Health 2009; 6: 790–804. 20101923 10.1123/jpah.6.6.790

[10] Kurimoto A , Awata S , Ohkubo T *et al*. Reliability and validity of the Japanese version of the abbreviated Lubben social network scale. Nihon Ronen Igakkai Zasshi 2011; 48: 149–157. 21778631 10.3143/geriatrics.48.149

[11] Ishikawa H , Nomura K , Sato M , Yano E . Developing a measure of communicative and critical health literacy: a pilot study of Japanese office workers. Health Promot Int 2008; 23: 269–274. 18515303 10.1093/heapro/dan017

[12] Tanaka T , Hirano H , Ohara Y , Nishimoto M , Iijima K . Oral frailty Index‐8 in the risk assessment of new‐onset oral frailty and functional disability among community‐dwelling older adults. Arch Gerontol Geriatr 2021; 94: 104340. 33529863 10.1016/j.archger.2021.104340

[13] Satake S , Shimokata H , Senda K , Kondo I , Toba K . Validity of total Kihon checklist score for predicting the incidence of 3‐year dependency and mortality in a community‐dwelling older population. J Am Med Dir Assoc 2017; 18: e1–552.e6. 10.1016/j.jamda.2017.03.013 28479274

[14] Folstein MF , Folstein SE , McHugh PR . ‘Mini‐mental state’. A practical method for grading the cognitive state of patients for the clinician. J Psychiatr Res 1975; 12: 189–198. 1202204 10.1016/0022-3956(75)90026-6

[15] Kobayashi S , Murakami K , Sasaki S *et al*. Comparison of relative validity of food group intakes estimated by comprehensive and brief‐type self‐administered diet history questionnaires against 16 d dietary records in Japanese adults. Public Health Nutr 2011; 14: 1200–1211. 21477414 10.1017/S1368980011000504

[16] Motokawa K , Mikami Y , Shirobe M *et al*. Relationship between chewing ability and nutritional status in Japanese older adults: a cross‐sectional study. Int J Environ Res Public Health 2021; 18: 1216. 33572969 10.3390/ijerph18031216 PMC7908427

[17] Suzuki F , Okamoto S , Miyagi S *et al*. Relationship between decreased mineral intake due to oral frailty and bone mineral density: findings from Shika study. Nutrients 2021; 13: 1193. 33916336 10.3390/nu13041193 PMC8066385

[18] Kimura M , Watanabe M , Tanimoto Y *et al*. Occlusal support including that from artificial teeth as an indicator for health promotion among community‐dwelling elderly in Japan. Geriatr Gerontol Int 2013; 13: 539–546. 22984985 10.1111/j.1447-0594.2012.00931.x

[19] Ringhof S , Stein T , Potthast W , Schindler HJ , Hellmann D . Force‐controlled biting alters postural control in bipedal and unipedal stance. J Oral Rehabil 2015; 42: 173–184. 25354425 10.1111/joor.12247

[20] Hirabayashi R , Edama M , Saito A , Yamada Y , Nawa R , Onishi H . Effects of clenching strength on exercise performance: verification using spinal function assessments. Sports Health 2022; 14: 404–414. 34053343 10.1177/19417381211014836 PMC9112714

[21] Kim EK , Lee SK , Choi YH *et al*. Relationship between chewing ability and cognitive impairment in the rural elderly. Arch Gerontol Geriatr 2017; 70: 209–213. 28214402 10.1016/j.archger.2017.02.006

[22] Yoshihara A , Takano N , Miyazaki H . The relationship between oral status and general health in subjects older than 65 according to selected items. J Dent Hith 2008; 58: 9–15.

