# Supplementary material for: Relationship between a gum‐chewing routine and oral, physical, and cognitive functions of community‐dwelling older adults: A Kashiwa cohort study
Source: Geriatr Gerontol Int. 2023 Dec 6;24(1):68–74. doi: 10.1111/ggi.14757 (PMC11503594; doi:10.1111/ggi.14757)
Supplement: Supplementary file 1 — DATA S1. Supporting Information. [file GGI-24-68-s001.docx]

**Supplementary Information**

**Supplementary Methods**

Each measurement method and references to oral examinations

*Gum-chewing routine*

The gum-chewing routine was assessed using the original self-administered questionnaire. Participants reported their gum-chewing habits on six levels as follows: 1. ‘‘multiple times per day,’’ 2. ‘‘five or more times a week,” 3. ‘‘a few times a week,” 4. ‘‘a few times a month,” 5. “not at all,” and 6. “used to eat but no longer.’’ The participants who answered “1” to “4” were asked how many minutes a week they chewed gum. As the median chewing time of those with a gum-chewing habit was 30 min, we classified those with a routine of chewing gum for more than 30 min as the gum-chewing routine group.

*Basic attributes*

*Body mass index (BMI)*

The BMI was measured using InBody 430 software. Because some participants could not use InBody 430, they were asked to self-report their weight, which was combined with their height measured on the day of the study to obtain the BMI by calculation.

*Frequency of visits to the dentist*

The frequency of visits to the dentist was converted into an ordinal scale using a self-administered questionnaire, where respondents were asked to choose “1. at least once every 3 months,” “2. at least once every 6 months”, “3. at least once a year”, “4. when there are symptoms, such as toothache,” or “5. rarely “^1^.

*Oral status*

Professionals, such as dentists or dental hygienists, measured oral statuses.

*Number of natural teeth/ functional teeth*

The number of natural and functional teeth was assessed by dentists or dental hygienists using the Zsigmondy–Palmer style^2^. Almost all Japanese dentists and dental hygienists use this style, suggesting that their calibration for dental diagnoses is sufficient. In this study, dental pontics for bridges, implants, and removable denture prostheses were also counted as functional teeth.

*Tongue coating index (TCI)*

The participant’s oral filths were measured using the TCI^3^. After categorizing the tongue into nine sections, the adhesion of the tongue coating on each section was evaluated at three levels (0, 1, or 2) and quantified as the TCI=Total score (0–18)/18×100(%). The measurers were given explanations of the three levels of adhesion in advance using photographs to avoid errors among the measurers.

*Maximum occlusal pressure*

The maximum occlusal force was measured using a pressure-sensitive film (Dental Pre-scale II; GC, Tokyo, Japan) and an analytical instrument (Byte Force Analyzer; GC)^4,5^. The participants were asked to bite a pressure-sensitive film with the maximum force for 3 s.

*Chewing ability*

Chewing ability was measured using color-changing chewing gum (XYLITOL; Lotte, Tokyo, Japan), which changed from green to red upon chewing. The participants were instructed to chew gum for 60 s at a rate of once per second. Additionally, we evaluated the red photo-genesis of the gum using a colorimeter (Color Reader CR-13; KONICA MINOLTA, Tokyo, Japan)^6^. Colorimeter measurements were performed at three locations on the same gum, and the average value was used. Other visual evaluations of gum color were performed using a 10-point color scale.

*Articulatory oral motor skill (“pa,” “ta,” and “ka” times/s)*

Oral diadochokinesis (“pa,” “ta,” and “ka”) was used^7^. Participants were asked to articulate each syllable repetitively as quickly as possible for 5 s. The syllables enabled the assessment of the following three major articulatory organs: the lips, tongue tip, and tongue dorsum. Articulation counts were measured using a digital counter (TKK-3351; Takei Scientific Instruments Co., Ltd., Niigata, Japan), and oral diadochokinesis was calculated separately for each syllable as articulation count per second.

*Maximum tongue pressure*

Tongue pressure was measured using a handheld balloon probe and manometer (JMS tongue pressure measurement instrument; GC)^8^. Participants were asked to place the balloon on the anterior part of the palate and raise their tongues to compress it onto the palate as forcefully as possible. The participants were asked to compress the balloon for 7 s. Three measurements were obtained, and the average value was used.

*Oral moisture*

Oral moisture was measured using a moisture-checking device (Mucus; Life, Saitama, Japan)^9^. The sensor was placed vertically approximately 10 mm from the tip of the tongue at constant pressure. The measurements were taken three times in succession, and the average value was obtained.

*Subjective measures*

Regarding subjective assessments, difficulties in eating and swallowing and experience of dry mouth were evaluated using the oral frail index-8 questionnaire with the questions as follows^1^: “Do you have any difficulties eating hard foods compared to 6 months ago: Yes or No?”, “Have you choked on your tea or soup recently: Yes or No?”, and “Do you frequently experience having a dry mouth: Yes or No?”

*Oral Frailty*

Oral frailty was defined as three or more of the following six items: (i) the number of natural teeth; (ii) chewing ability; (iii) articulatory oral motor skill for “ta;” (iv) tongue pressure; (v) subjective difficulty in eating tough foods; and (vi) subjective difficulty in swallowing^10^.

The numbers were established using the 2018 paper (Oral frailty as a risk factor for physical frailty and mortality in community-dwelling elderly) as a reference. Furthermore, (i) was determined based on the number of teeth being less than 20. For (ii), the cutoff value was less than the lower quintile of the α* value for each sex. The cutoff values were less than 18.8 and 17.87 for male and female individuals, respectively. In (iii), the cutoff value was also set at less than the lower quintile for each sex. The cutoff values were less than 5.8 and 5.9 for male and female individuals, respectively. The cutoff value for (iv) was also less than the lower quintile for each gender. The cutoff values were less than 26.8 and 26.0 for men and women, respectively. In (v), those who answered “yes” to whether they had less difficulty in eating hard foods than 6 months earlier were considered applicable. In (vi), the respondents who answered “yes” to the question, “Do you sometimes choke on tea or soup? These participants were defined as those who answered “yes” to the question, “Do you have difficulty in eating solid foods compared to 6 months ago? This condition is described in the supplemental section.

*Frailty*

Participants were asked to respond to a self-administered Kihon checklist questionnaire to measure frailty^11^. Eight or more items indicated frailty.

*Grip strength*

The grip strength was measured using a grip strength meter. The participants were measured in the standing position^12^. Two measurements were taken for the dominant hand, and the higher value was used for the measurement site.

*One-leg stand with eyes open for 60 s*

The patients were asked to stand with one leg open for 60 s with the dominant leg^13^; those who achieved less than 60 s were evaluated at that value, and those who reached 60 s or more were assessed as 60 s.

*Timed “Up and Go” (TUG) test*

The TUG test required participants to perform a series of actions, including getting up from a comfortable sitting position in a chair, walking 3 m away, turning around at a set-up cone, walking 3 m again, and sitting down deeply^14^. The participants were asked to perform a series of movements twice, and the shorter time was used for the evaluation.

*Normal gait speed and maximum gait speed*

Both normal and maximum gait speeds were measured at 5 m intervals. An 11 m gait lane was created, and the participants were asked to walk 5 m in the lane at two different gait speeds, normal and maximum, and the time taken was evaluated^15^. The time at the normal gait speed was measured once and evaluated. For maximum gait speed, two measurements were taken, and the shorter time was evaluated.

**Supplementary References**

1. Tanaka T, Hirano H, Ohara Y, Nishimoto M, Iijima K. Oral Frailty Index-8 in the risk assessment of new-onset oral frailty and functional disability among community-dwelling older adults. *Arch Gerontol Geriatr* 2021; 94: 104340.

2. Zsigmondy A. Grundzude einer praktischen Methode zur raschen und genauen Vormerkung der zahanarztlichen Beobachtungen und Operatione. *Dtsch Vjschr Zahnhk* 1861; 1: 209–211.

3. Shimizu T, Ueda T, Sakurai K. New method for evaluation of tongue-coating status. *J Oral Rehabil* 2007; 34: 442–447.

4. Miura H, Watanabe S, Isogai E, Miura K. Comparison of maximum bite force and dentate status between healthy and frail elderly persons. *J Oral Rehabil* 2001; 28: 592–595.

5. Horibe Y, Matsuo K, Ikebe K, *et al*. Relationship between two pressure-sensitive films for testing reduced occlusal force in diagnostic criteria for oral hypofunction. *Gerodontology* 2022; 39: 3–9.

6. Weich S, Burton E, Blanchard M, Prince M, Sproston K, Erens B. Measuring the built environment: validity of a site survey instrument for use in urban settings. *Health Place* 2001; 7: 283–292.

7. Sakayori T, Maki Y, Hirata S, Okada M, Ishii T. Evaluation of a Japanese “Prevention of long-term care” project for the improvement in oral function in the high-risk elderly. *Geriatr Gerontol Int* 2013; 13: 451–457.

8. Utanohara Y, Hayashi R, Yoshikawa M, Yoshida M, Tsuga K, Akagawa Y. Standard values of maximum tongue pressure taken using newly developed disposable tongue pressure measurement device. *Dysphagia* 2008; 23: 286–290.

9. Minakuchi S, Tsuga K, Ikebe K, *et al*. Deterioration of oral function in the elderly. *J Gerodont* 2016; 31: 81–99.

10. Tanaka T, Takahashi K, Hirano H, *et al*. Oral frailty as a risk factor for physical frailty and mortality in community-dwelling elderly. *J Gerontol A Biol Sci Med Sci* 2018; 73: 1661–1667.

11. Satake S, Shimokata H, Senda K, Kondo I, Toba K. Validity of total Kihon checklist score for predicting the incidence of 3-year dependency and mortality in a community-dwelling older population. *J Am Med Dir Assoc* 2017; 18: 552.e1–552.e6.

12. Fried LP, Tangen CM, Walston J, *et al*. Frailty in older adults: evidence for a phenotype. *J Gerontol A Biol Sci Med Sci* 2001; 56: M146–M156.

13. Drusini AG, Eleazer GP, Caiazzo M, *et al*. One-leg standing balance and functional status in an elderly community-dwelling population in northeast Italy. *Aging Clin Exp Res* 2002; 14: 42–46.

14. Beauchet O, Fantino B, Allali G, Muir SW, Montero-Odasso M, Annweiler C. Timed UP and Go test and risk of falls in older adults: a systematic review. *J Nutr Health Aging* 2011; 15: 933–938.

15. Furuna T, Nagasaki H, Nishizawa S, *et al*. Longitudinal change in the physical performance of older adults in the community. *J Jpn Phys Ther Assoc* 1998; 1: 1–5.

**Supplementary Figure 1.** Flow chart of participants

**
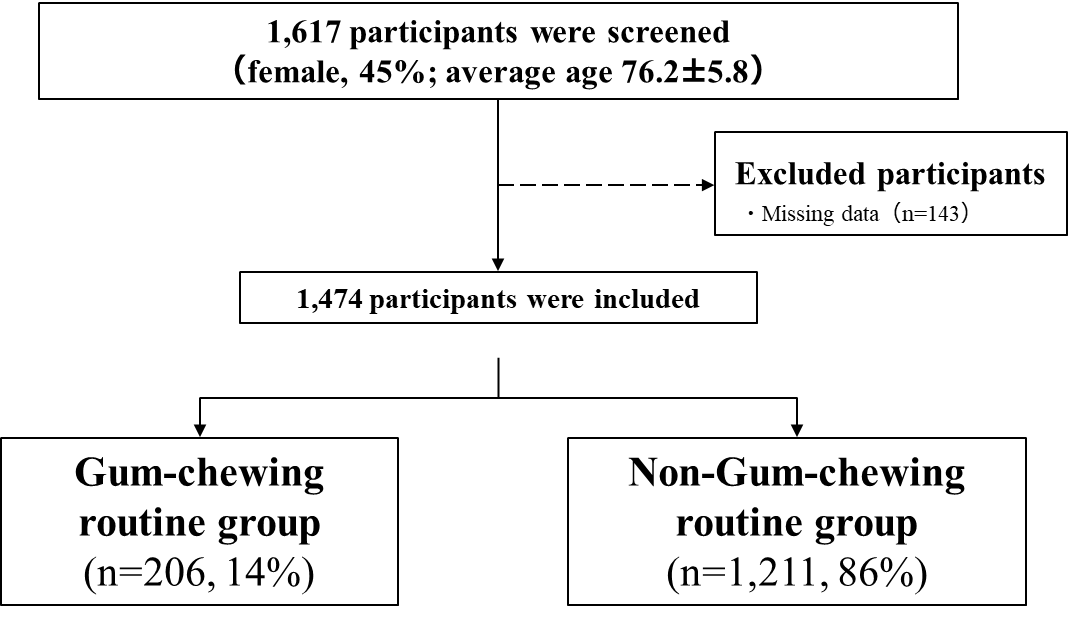
**

**Table S1.** Applicants for each oral frailty item between the gum-chewing routine and non-gum-chewing routine groups

| Factors  (Dependent variables) | Overall  (n = 1,474) | Gum-chewing routine group  (n = 206) | Non-gum-chewing routine group  (n = 1,268) | Non-gum-chewing routine group | Gum-chewing routine group | |
| --- | --- | --- | --- | --- | --- | --- |
|  |  |  |  | OR | Crude OR (95% CI) ^†^ | aOR (95% CI) ^‡^ |
| Oral frailty | 205 (13.9%) | 17 (8.3%) | 188 (14.8%) | 1.00 (reference) | 0.52 (0.31–0.87) ^*^ | 0.58 (0.34–0.99) ^*^ |
| (i) Less number of remaining teeth, n | 348 (23.6%) | 19 (9.2%) | 329 (25.9%) | 1.00 (reference) | 0.29 (0.18–0.47) ^**^ | 0.31 (0.19–0.50) ^**^ |
| (ii) Low chewing ability, n | 292 (19.8%) | 36 (17.5%) | 256 (20.2%) | 1.00 (reference) | 0.84 (0.57–1.23) | 0.87 (0.59–1.29) |
| (iii) Low articulatory oral motor skill for  “ta,” n | 226 (15.3%) | 25 (12.1%) | 201 (15.9%) | 1.00 (reference) | 0.73 (0.47–1.14) | 0.81 (0.51–1.27) |
| (iv) Low tongue pressure, n | 281 (19.1%) | 26 (12.6%) | 255 (20.1%) | 1.00 (reference) | 0.57 (0.37–0.89) ^*^ | 0.61 (0.39–0.96) ^*^ |
| (v) Subjective difficulty in eating tough foods, n | 265 (18.0%) | 23 (11.2%) | 242 (19.1%) | 1.00 (reference) | 0.53 (0.34–0.84) ^**^ | 0.55 (0.34–0.87) ^*^ |
| (vi) subjective difficulty in swallowing, n | 312 (21.2%) | 48 (23.3%) | 264 (20.8%) | 1.00 (reference) | 1.16 (0.81–1.64) | 1.18 (0.83–1.68) |
| *Notes*: OR, odds ratio; aOR, adjusted odds ratio; CI, confidence interval.  Data are shown as the number of participants (percentages).  ^†^ Results of binomial logistic regression analysis when with/without gum-chewing routine was used as a covariate, and each factor was used as a dependent variable (* p < 0.05 and ** p < 0.01).  ^‡^ Adjusted model included as covariates: age, sex, body mass index, living arrangements, education duration, number of medications, Lubben Social Network Scale-6 scores, Communicative and Critical Health Literacy scale scores. For only analysis of oral status and oral frailty, the frequency of visits to the dentist was also used as a covariate. (* p < 0.05 and ** p < 0.01). | | | | | | |
